# Hyalinizing Granuloma: An Unusual Case of a Pulmonary Mass

**DOI:** 10.1155/2010/984765

**Published:** 2010-06-14

**Authors:** Viviane Brandão, Edson Marchiori, Gláucia Zanetti, Guilherme Abdalla, Nina Ventura, Carolina Lamas Constantino, Mariana Leite Pereira, Pedro Martins, Rodrigo Canellas, Antonio Muccillo, Romulo Varella de Oliveira

**Affiliations:** ^1^Department of Radiology, Rio de Janeiro Federal University, CEP 21941.913 Rio de Janeiro, Brazil; ^2^Department of Radiology, Fluminense Federal University, CEP 24220.071 Rio de Janeiro, Brazil

## Abstract

We describe the case of pulmonary hyalinizing granuloma in a 34-year-old asymptomatic man who presented with a pulmonary nodule apparent by chest radiography and computed tomography (CT). He had a history of previous treatment for tuberculosis. His laboratory data were normal. Bronchoscopy and CT-guided percutaneous transthoracic fine needle aspiration cytology were inconclusive. The diagnosis was revealed after the histopathological examination of an open lung biopsy.

## 1. Introduction

Pulmonary hyalinizing granuloma (PHG) is a rare benign lung disease characterized by fibrosing nodules, consisting of either unilateral or bilateral central whorled deposits of lamellar collagen hyaline [1]. The exact etiology of this condition is unknown [2], although an exaggerated immune response to the antigenic stimuli by infection or autoimmune process has been suggested [3, 4]. The clinical presentation of PHG ranges from vague chest symptoms to no symptoms at all. Chest radiography and computed tomography will reveal single or multiple well-defined nodules with a random distribution in persons with PHG. There are few complications with PHG and the patients have an excellent prognosis [5]. Because the symptoms are mild, most of the lesions are incidental radiological findings and are not initially correctly diagnosed. PHG should hence be included in the differential diagnosis of patients with pulmonary nodules.

## 2. Case Presentation

A 34-year-old man without symptoms was referred to our hospital to clarify an abnormal chest radiograph showing a nodular pulmonary lesion. He was currently without symptoms, but had been treated two years prior for tuberculosis after presenting with mild fever, nonproductive cough, and a five-pound weight loss. There were no comorbidities and the patient denied a history of smoking. Physical examination revealed no abnormalities.

**Figure 1:**
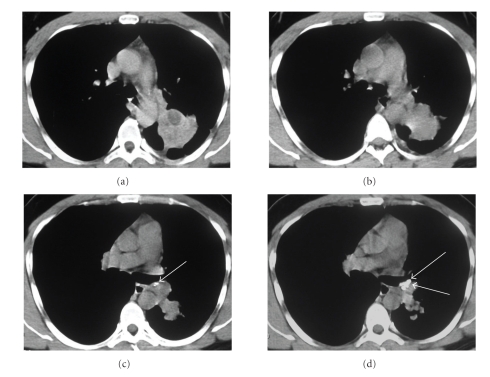
HRCT scans with mediastinal window (a)–(d) demonstrated a mass with irregular borders, measuring about 6 cm in diameter, with pleural extension and thickening of adjacent pleura, located in the upper segment of the left lower lobe. The mass was heterogeneous with hypodense areas. Amorphous calcifications were also present in the mass (c, d) (arrows).

The chest radiography revealed a well-circumscribed mass in the left lung. CT scans demonstrated a mass with irregular borders, measuring approximately 6 cm in diameter, located in the upper segment of the lower lobe of the left lung. The mass was heterogeneous with hypodense areas and amorphous calcifications. No mediastinal lymphadenopathy was observed (Figure 1). 

Pulmonary function tests revealed a mild obstructive ventilatory disturbance. Laboratory data, including rheumatoid factor and antinuclear body, were normal. Bronchoscopy was normal and mycobacterial and fungal cultures of bronchoalveolar lavage fluid were negative. Cytological evaluation was negative for malignant cells. CT-guided percutaneous transthoracic fine needle aspiration cytology of the pulmonary lesion was nondiagnostic. There was no evidence of bacterial or fungal organisms in the collected sample.

**Figure 2:**
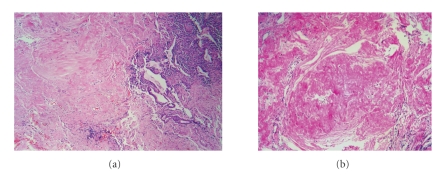
(a) Marked changes in lung architecture determinated by deposition of hyaline tissue masses accompanied by sparse lymphocytic infiltrate that compresses and distorts the remaining bronchioles (hematoxilin-eosin stain; original magnification, x40). A higher magnification (b) demonstrates that the mass is composed by hypocellular collagen lamellae (hematoxilin-eosin stain; original magnification, x100).

An open lung biopsy revealed a mass in the upper segment of the lower lobe of the left lung and fibrous exudate, which was adhered to the pleural surface. The histopathological findings mainly consisted of deposition of hyaline tissue masses accompanied by sparse lymphocytic infiltrate (Figure 2). There was no histological evidence for infectious agents such as TB and fungal organisms. These features were consistent with a diagnosis of pulmonary hyalinizing granuloma. 

The patient received no subsequent specific treatment and he remained asymptomatic. There was no increase in the size of the mass as of the last outpatient followup, which was performed two years after diagnosis.

## 3. Discussion

PHG is a rare fibrosing nodular disease known to affect adults between the ages of 19 and 77 years. The mean age of person afflicted with the disease is 44 years [1] and there is no sex predilection or race predominance [2, 6]. The clinical symptoms of PHG are mild and nonspecific, and may include cough, fever, fatigue, dyspnea, pleuritic chest pain, sinusitis, and pharyngitis. Several patients are completely asymptomatic with lesions only found through routine screening examinations [1, 5, 7].

The etiology of PHG remains unclear. It has been proposed that the nodules may represent an abnormal immune response to infectious agents, such as tuberculous bacilli and histoplasma organisms, or autoimmune process, since many immune-mediated diseases appear in concordance with PHG [1, 2, 4, 5, 7, 8]. There have been reports of patients who were previously exposed to fungal or mycobacterial diseases prior to contracting PHG [6]. Additionally, patients presenting with immunologic abnormalities and elevated serum markers of progressive autoimmune diseases, such as antinuclear antibodies, rheumatoid factor, and positive antiglobulin tests may be more prone to developing PHG [8]. Sclerosing mediastinitis, retroperitoneal fibrosis, rheumatoid arthritis, posterior uveitis, Sjogren's syndrome, hemolytic anemia, and other diseases are often associated with PHG [1]. It has been hypothesized that all of these conditions may essentially represent the same reactive response of an immunologic mechanism triggered by infectious agents [9]. Associations of PHG with lymphoproliferative disorders, such as lymphoma and Castleman's disease, have also been described [3, 9].

Chest radiography and CT findings show solitary or, more often, multiple randomly distributed, unilateral or bilateral nodules and/or masses with well-defined borders. The nodules may be present with or, more commonly, without calcification and are typically focal and irregular [10]. The calcified masses are more often multiple and bilateral [10]. Though rare, cavitation has been reported [10, 11]. Nodule size ranges from several millimeters to 15 cm in diameter, with an average diameter of 2 cm [9]. Adenopathy is usually not associated with this entity [1].

Differential diagnosis in PHG includes malignancy (metastatic or primary), infection (septic emboli, tuberculosis, histoplasmosis, or other fungal infections), amyloidosis, rheumatoid nodules, Wegener's granulomatosis, sarcoidosis, lymphomatoid granulomatosis, and plasma cell granuloma [3, 6–8, 10, 11]. When calcified, sarcomatous metastases (e.g., osteosarcoma, chondrosarcoma and giant cell tumor) and carcinomatous metastases (e.g., mucin-producing adenocarcinomas, thyroid cancer, and choriocarcinoma) should be included in the differential diagnosis [10]. The aforementioned masses can be differentiated by their clinical, biological, bacteriological, and histopathological characteristics [8]. 

F-18 fluordeoxyglucose positron emission tomography (FDG-PET/CT) can reveal increased metabolic activity in PHG lesions [12]. However, an accurate diagnosis of PHG can only be made with histopathological examination [8]. Microscopically, the lesions are well-circumscribed and are characterized by a dense network of concentric hyalinized collagen in the center of perivascular lymphoplasmacytic infiltrate that rarifies in the center of the nodule [5]. The collagen is deposited in ropy, whorled collagen bundles that are separated by clear spaces [8]. Congo red stain with polarization and crystal violet stain can be positive for amyloid, and the diagnosis of amyloidosis must be considered. Amyloidosis can be ruled out using electron microscopy because in PHG the hyaline lamellae consist of eletrondense, compact, amorphous material, which is substantially different from fibrillar amyloid lesions [4, 8]. 

The prognosis for patients with PHG is generally excellent with no significant impact on longevity. Single lesions tend to be stable and resection is often curative. Some patients with multiple lesions may show progressive enlargement of nodules and increased dyspnea [1, 5]. There is no definitive treatment for multiple nodules, although successful resolution of the nodules with the administration of glucocorticoids has been reported [1, 5, 12, 13]. FDG-PET/CT may demonstrate a decrease in metabolic activity after corticosteroid treatment [12]. One case of recurrence after resection was described [14]. 

In conclusion, PHG should be considered in the differential diagnosis of pulmonary nodules or masses, even when they are cavitary or contain calcifications.
